# 3D-visualization of segmented contacts of directional deep brain stimulation electrodes via registration and fusion of CT and FDCT

**DOI:** 10.1186/s41824-024-00208-6

**Published:** 2024-06-14

**Authors:** Fadil Al-Jaberi, Matthias Moeskes, Martin Skalej, Melanie Fachet, Christoph Hoeschen

**Affiliations:** 1 Chair of Medical Systems Technology, Institute for Medical Technology, Faculty of Electrical Engineering and Information Technology, Otto von Guericke University Magdeburg, Universitätsplatz 2, 39106 Magdeburg, Germany; 2 Research Department, Missan Oil Company, Iraqi Ministry of Oil, Baghdad, Iraq; 3 Institute of Biometry and Medical Informatics, Medical Faculty, Otto von Guericke University Magdeburg, Leipziger Str. 44, 39120 Magdeburg, Germany; 4 Neuroradiology, Medical Faculty, Martin Luther University Halle-Wittenberg, Ernst-Grube-Straße 40, 06120 Halle, Germany

**Keywords:** Deep brain stimulation, Medical image fusion, Multimodal medical image registration, 3D rigid registration, Image-guided intervention

## Abstract

**Objectives:**

3D-visualization of the segmented contacts of directional deep brain stimulation (DBS) electrodes is desirable since knowledge about the position of every segmented contact could shorten the timespan for electrode programming. CT cannot yield images fitting that purpose whereas highly resolved flat detector computed tomography (FDCT) can accurately image the inner structure of the electrode. This study aims to demonstrate the applicability of image fusion of highly resolved FDCT and CT to produce highly resolved images that preserve anatomical context for subsequent fusion to preoperative MRI for eventually displaying segmented contactswithin anatomical context in future studies.

**Material and methods:**

Retrospectively collected datasets from 15 patients who underwent bilateral directional DBS electrode implantation were used. Subsequently, after image analysis, a semi-automated 3D-registration of CT and highly resolved FDCT followed by image fusion was performed. The registration accuracy was assessed by computing the target registration error.

**Results:**

Our work demonstrated the feasibility of highly resolved FDCT to visualize segmented electrode contacts in 3D. Semiautomatic image registration to CT was successfully implemented in all cases. Qualitative evaluation by two experts revealed good alignment regarding intracranial osseous structures. Additionally, the average for the mean of the target registration error over all patients, based on the assessments of two raters, was computed to be 4.16 mm.

**Conclusion:**

Our work demonstrated the applicability of image fusion of highly resolved FDCT to CT for a potential workflow regarding subsequent fusion to MRI in the future to put the electrodes in an anatomical context.

**Supplementary Information:**

The online version contains supplementary material available at 10.1186/s41824-024-00208-6.

## Introduction

### Deep brain stimulation

With neurodegenerative disorders such as Parkinson’s Disease (PD) being increasing in incidence and prevalence worldwide their socioeconomic importance rises accordingly. According to the “Global Burden of PD” study there was a doubling in prevalence from 1990 until today. Thus the prevalence is projected to reach 12 million by 2040 (Feigin et al. 2019). The main features of PD are bradykinesia, postural instability and rest tremor. Psychiatric symptoms such as delusion or hallucinations may arise as well (Bloem et al. 2021).

**Fig. 1:**
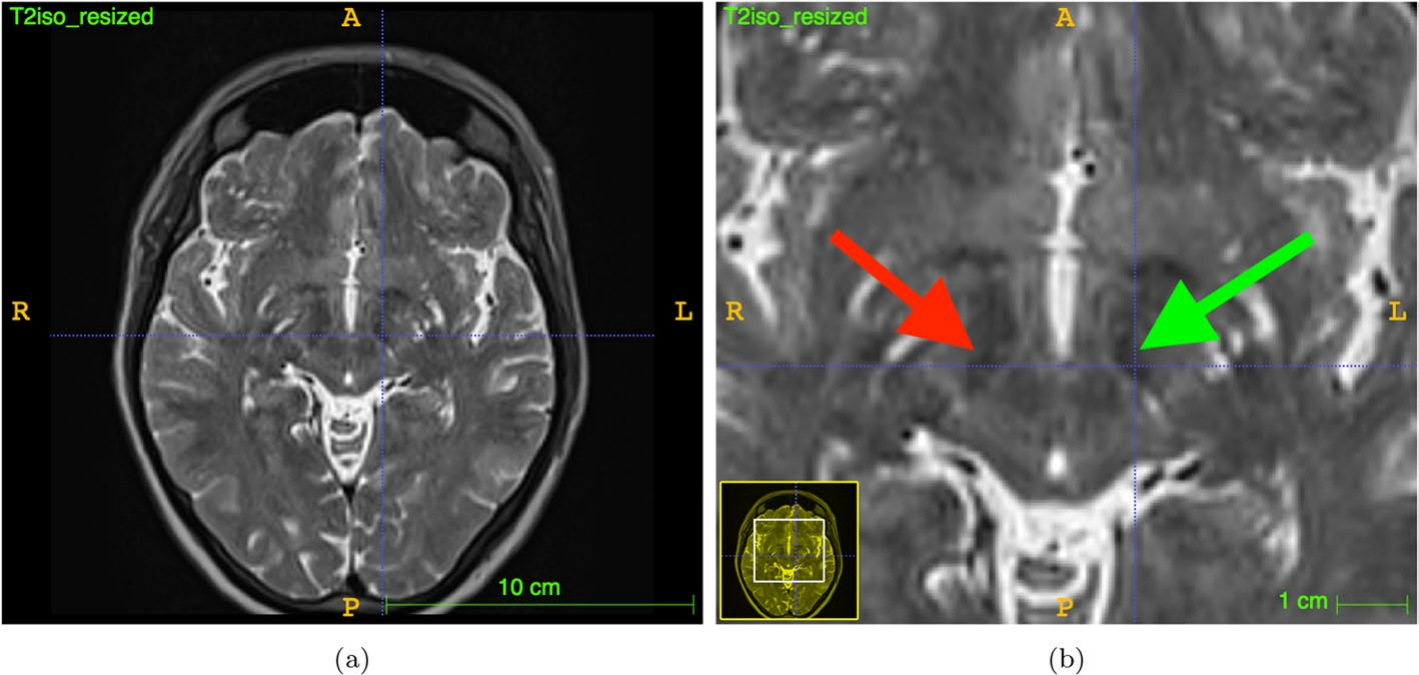
**a** Preoperative T2-weighted MRI, and **b** characteristic hypointensities depicting right and left STN on preoperative T2-weighted MRI (right: red arrow, left: green arrow)

These symptoms may be lowered by oral dopamin-agonistic drug treatment yet not often fully eliminated (Hayes 2019). Generally neurodegeneration leads to different patterns of electric acitivity in certain brain regions, which leads to the idea of altering the pathological activity via electric stimulation. In PD the most important deep brain target is the subthalamic nucleus (STN, Fig. 1).

This treatment is called Deep Brain Stimulation (DBS) and is increasingly applied nowadays and mainly used to treat movement disorders such as PD, dystonia and tremor (Lozano et al. 2019) or psychiatric conditions like treatment-resistant depression (TRD) though current research indicates promising results in Alzheimer’s Disease as well (Kuhn et al. 2010). It generally involves the invasive placement of an electrode inside subcortical brain targets to alter their electric activity in a controlled manner (Hammond et al. 2008; García et al. 2013). DBS is capable of improving motor and non-motor symptoms in PD (Koivu et al. 2022) and shows some potential for the treatment of other conditions (Reinacher et al. 2017; Bittlinger and Müller 2018). The DBS device itself consists of a lead, a subcutaneous extension, an electrode as well as a pulse generator (Flemming and Wingender 2010). Current electrode models are capable of steering the stimulating electric field which is known as directional DBS (dDBS). Knowledge of the exact location of the stimulation target as well as the location of the electrode is mandatory to implant and eventually program the device (Schmidt et al. 2022). Common clinical workflows consist of preoperative computed tomography (CT) imaging for stereotactic guidance as well as postoperative CT-imaging for rule out of bleeding and determination of the electrode orientation while the visualization of tiny orientation markers of the electrode is a domain of intraoperative X-ray imaging (Schmidt et al. 2022; Vickers 2017).

### Imaging in deep brain stimulation

The implantation procedure often involves multimodal imaging and in some cases image fusion of stereotactic CT and MRI to identify the target in a 3D-manner and obtain information of physical coordinates in stereotactic space (Schmidt et al. 2022). Prior to surgery, MRI would be performed according to a center-specific planning protocol. Intraoperative CT is usually performed after mounting a stereotactic frame onto the patient. Subsequent registration of MRI to stereotactic CT and normalization to brain atlas space let the surgeon visually locate the target of stimulation. An optimal trajectory for implantation (e.g. least likely to hurt vessel) is estimated and stereoteactic coordinates are retrieved. On the stereotactic frame a guiding tubes position is adjusted according to the trajectory defined beforehand. Following implantation may involve one or two hemispheres which is usually accompanied by stereotactic imaging confirmation employing image fusion of intraoperative CT or 3D-X-Ray to MRI (Schmidt et al. 2022; Egger et al. 2022). Stimulation settings are usually programmed using microelectrode recordings and clinical examinations (Koeglsperger et al. 2019). However leads may show deviations from their intended implantation orientation (Dembek et al. 2019). On one hand, this may be due to biological factors e.g. edema or brain shift as well as pneumencephalon and/or hemorrhage (Dembek et al. 2019; Shamji and Isaacs 2008). On the other hand, mechanical factors e.g. unintended application of force during lead implantation and fixation respectively are suspicious to mediate lead deviations even more than biological factors (Schmidt et al. 2022). Ultimately, a combination of the aforementioned issues seems likely making it quite impossible to predict time or magnitude of lead deviation (Rau et al. 2021). Hence the localization of electrically active contacts might be no longer corresponding to the underlying anatomical context of presurgical MRI when programming the electrode (Merola et al. 2020). 3D-visualization of the lead markers and contacts orientation in anatomical context could shorten the time for postoperative adjustment of stimulation parameters (Schmidt et al. 2022). Since electrode orientation is stable from a certain point after implantation (Dembek et al. 2021) high-resolution 3D visualization of the electrodes could be a measure of precise determination of electrode orientation not only during but also after implantation.

### Flat detector

**Fig. 2:**
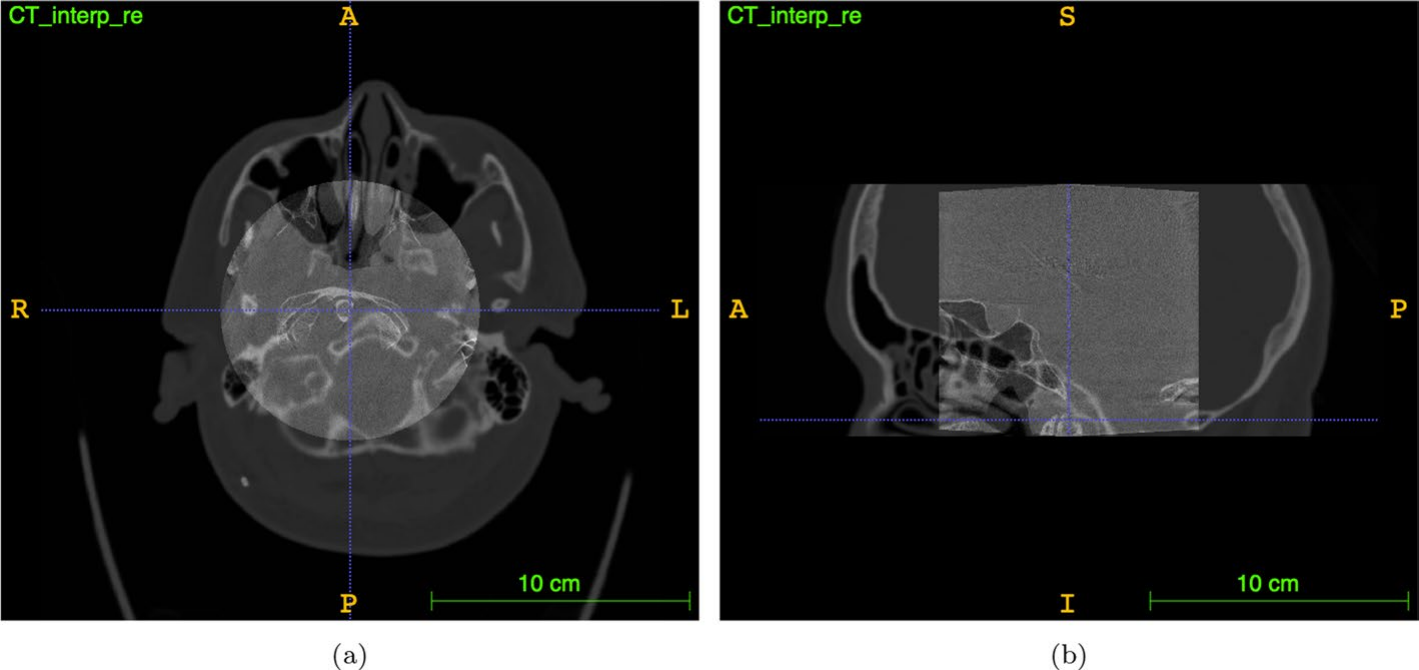
Transparent overlay of unregistered data: **a** image shows axial slice and **b** image shows mid-sagittal slice of DCTm and MS-CT in one patient (after interpolation, CT is cut along z for computational purposes)

The basis for flat detector computed tomography (FDCT) lays in a combination of a rotating X-Ray tube and a flat panel detector that allow for volumetric data aquisition. The projection data is reconstructed three-dimensionally with respect to Cone-Beam geometry (Orth et al. 2008). Since the material properties of the electrodes and their dimensions require less contamination of the image data by scattering or metal artifacts and a higher spatial resolution than with multislice CT, the use of FDCT protocols tailored to small implants seems reasonable. Hence spatially high resolved images were obtained using the “22s DynaCT micro head” (©Siemens, Erlangen, Germany) (DCTm) protocol which produces a cylindric volume featuring voxel size of 0.1965 mm within a 512 pixel matrix and 16-bit gray scale resolution. The resulting volume is characterized through a narrow field-of-view (FOV) leading to a cut-out aspect of intracranial high-contrast objects (e.g. bone, metal). In general, matching the FOV to the anatomical region of interest is important because it reduces patient dose and improves image contrast by reducing scattered radiation (Orth et al. 2008). Regarding the electrodes the FOV should be chosen to represent the volume-of-interest (VOI) within the skull (Fig. 2).

### Image registration

Image registration is the process of matching a moving image to the coordinate system of a fixed image and can be viewed as the foundation for image fusion. It can be done automatically via numerical optimization of transformation parameters with respect to a measure of dissimilarity between the images (Papenberg et al. 2011; Bhattacharya and Das 2011). Moreover, manual matching of control point pairs may also deliver the correct parameters (Maurer et al. 1997). In the rigid case, meaning neither shear nor scaling are apparent, there are six parameters that describe translation and rotation between the images. Automatic methods work indirect as they iteratively search for the best solution and thus can get stuck in a local minimum of the similarity function. This risk increases with higher initial displacement and less corresponding features being displayed (Papenberg et al. 2011). Manual registration methods tend to be more robust than as they deliver a direct solution without the risk of converging to a local minimum yet being critically dependent on user input (Papenberg et al. 2011).

## Material and methods

### CT and FDCT image data acquisition

Postulating FDCT and MRI do not share enough anatomical information for a direct fully automated registration it was decided to register the FDCT to the CT. The resulting dataset could be fused to MRI via established CT-MRI image fusion techniques in future work. Hence we used a retrospectively collected data set from clinical routine from fifteen patients ($n=15$) that underwent bilateral dDBS implantantion surgery. Rotational fluoroscopy and computation of DCTm were performed within the first week after surgery, where a CT was performed right after surgery leading to a mean time difference between examinations of 4.5 days. The mean age of patients evaluated was 53 years, with 8 females and 7 males included in the study. For each patient rotational fluoroscopy was performed on an *ArtisQ* multi-purpose x-ray-system. As pointed out in the introduction highly resolved FDCT (hrFDCT) was computed using *syngo DynaCT micro head* (both *©Siemens, Erlangen, Germany*)). The settings for DCTm were an anode voltage of 116–119 kV with a tube current ranging from 258 to 274 mA and a pixel matrix of size $512 \times 512 \times 497$ with an isotropic voxel size of $0.1965\times 0.1965\times 0.1965\,\hbox {mm}$.

The conventional (multislice) CT was performed with a *SOMATOM Definition AS+* ( ©Siemens, Erlangen, Germany) employing an anode voltage of 120 kV and a tube current of 370 mA. The CT provided 197–253 slices respectively with an image size of $512 \times 512$. Due to the FOV being adjusted by a radiological technician, the system yielded voxel sizes from $0.4{-}0.6\times 0.4{-}0.6\times 0.75\,\hbox {mm}$.

As stated previously DCTm is considered to represent a 3D-ROI in CT because DCTm does not display the skull to it’s full extent.

### Computing resources and toolkits

All methods presented in this paper for image registration, analysis and evaluation are based on the open-source software Insight Toolkit (ITK) (Beare et al. 2018; Yaniv et al. 2018), and using programming language Python. ITK is an open-source medical imaging research toolkit, primarily used for segmenting and registering medical images. The computing resource used a Bluechip 64-bit Windows 10 Pro operating system with an Intel Core i7-11700 processor, 2.50 GHz and 32 GB RAM.

### Image processing workflow

Our approach entails a semi-automated workflow for multimodal 3D image registration, utilizing both CT and DCTm images. The input image files were in format DICOM (Digital Imaging and Communications in Medicine), while output and subsequent analysis were conducted in MetaImage (MHA) format (refer to “Medical Image Formats” section for more details). The registration workflow primarily involved rigid image registration (RIR), the term “rigid” in rigid image registration refers to the assumption that the transformation applied to one image to align it with another is limited to rotations and translations, without any deformation or scaling. In other words, the relative positions and orientations of the objects in the images may change, but their shapes and sizes remain constant.

In our case, RIR is used for aligning different imaging modalities in DBS application, e.g. for aligning postoperative FDCT and CT images. Further details on the registration methods have been provided in this paper, as elucidated in “Taxonomy of registered images” section.

During the visualization of both images, the task of identifying corresponding points between these modalities necessitated the utilization of key anatomical landmarks. The recognition of these anatomical landmarks demanded specialized expertise, because the CT and DCTm images differ in terms of FOV, slice thickness, and pixel spacing. Further details are explained in “Anatomical landmarks used for registration” section. The workflow included a simultaneous reading and visualization of CT and DCTm images using SimpleITK toolkits and Python, employing parallel windowing.

Following this, manual initialization was executed based on user input, discerning three points within each CT image that corresponded to the DCTm image via anatomical landmarks. Subsequently, the components of registration were defined, encompassing transformation, interpolation, similarity metrics, and optimization. To understand this in detail, it is illustrated in Fig. 5, illustrating the framework of our semi-automated registration approach. Post completion of registration, the outcomes were saved in MHA format. Our registration procedure entailed assigning one image as the fixed image, while the others were designated as moving images. After manual initiation through user input involving anatomical landmarks, the moving image underwent the processes outlined in “Registration framework” section. Initially, a CT image served as the fixed image, and a DCTm image functioned as the moving image. Subsequently, we reversed this procedure, utilizing the DCTm image as the fixed image and the CT image as the moving image. This enabled us to generate supplementary outcomes for analysis, as depicted in Fig. 6.

The evaluation methods for image registration within this study encompass qualitative and quantitative techniques. In the realm of qualitative evaluation, diverse methods were applied, including linked cursors, checkerboard patterns, and image fusion using alpha blending. In the checkerboard pattern approach, prior to combining two images, the moving image had to be resampled to ensure that both images occupied the same spatial domain (Beare et al. 2018; Yaniv et al. 2018). Additionally, the intensity levels were rescaled to ensure uniform intensity ranges. Specifically, they were mapped to a range of 0 to 255, contingent on the desired windowing specifications for the CT and DCTm images. For quantitative evaluation, the pivotal approach employed was the analysis of target registration errors (TRE) in the context of multimodal image registration. The TRE serves as a metric for gauging the accuracy of the registration process. It quantifies the discrepancy between the coordinates of the corresponding points in the registered images. In other words, it indicates how well the registered images match in terms of spatial alignment of anatomical or other features. The closer the value of TRE is to zero, the better the image registration result. When the TRE value is zero, it means that the images are identical. TRE is usually calculated as the Euclidean distance between the corresponding points in the images before and after registration. It helps to evaluate the effectiveness of the registration algorithm and gives an indication of the quality of the alignment obtained. A low TRE value indicates better registration accuracy. This involves computing the TRE by comparing the transformation of fixed and moving points, represented by $T_f^m$ and $^fp,^mp$ respectively, using the formula:

1 $$\begin{aligned} TRE =|T_f^m(^fp) - ^mp| \end{aligned}$$

The pairs of equivalent points in the fixed and moving coordinate systems are acquired, but they are not involved in the registration process. The transformation estimated by the registration is then applied to the points in the fixed coordinate system and the distance between the transformed points and their actual locations in the moving coordinate system is used to calculate the TRE.

### Medical image formats

As shown in Fig. 5, we used DICOM as input and MHA as output format for the multimodal image registration framework. As a standard format used in clinical and hospital settings for storing, transmitting, and sharing medical images and associated patient data, we acquired a retrospectively collected data set as DICOM files. It is a complex file format that includes both image data and metadata, including patient information, acquisition parameters, image annotations, and other clinical details. We have multiple DICOM files in our dataset, meaning each slice is stored in a separate DICOM file. The process of reading and writing is not straightforward and flexible when it comes to image registration and further analysis. In our case, MHA files are a structured format for storing and analyzing medical images that supports multidimensional data, which is straightforward and flexible. The MHA file contains only image data and header information that describes the image size, pixel spacing, origin, and data type.In this work, Python was used as the programming language to read, further process and write the images. MHA is an open-source file format, and it is supported by multiple open-source medical imaging software packages and libraries, including the ITK for Segmentation and Registration Toolkit. It is for these reasons that MHA files are more commonly used in research applications, particularly when segmenting and registering images.

### Taxonomy of registered images

**Fig. 3:**
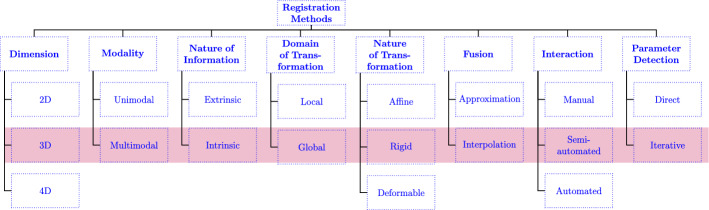
Taxonomy of medical image registration (Alam et al. 2016)

Our objective in the context of medical image registration taxonomy is to elucidate the details of our registration approach. Figure 3 illustrates the specific type of registration in various aspects, which include the following categories: Dimension, modality, nature of information, domain, nature of transformation, fusion, interaction, and the parameter detection that have been used in this work. The following methods (Fig. 3) will be described in further detail for this work.

A distinction can be made between extrinsic and intrinsic image-based registration. Extrinsic techniques are the imaged space containing extraneous objects, intrinsic techniques such as those based on image information generated by the patient. Since the registration procedures used contain patient-related image information, the procedures in this work can be classified as intrinsic techniques, where the anatomical landmarks are usually used semi-automatically (initialized by the user). The registration is called semi-automatic if it includes user initialization, user steering (correction), or both. Image interpolation techniques are a widely used process in medical imaging. High-resolution slice sequences of organs or tissues are acquired using CT, MRI or other imaging modalities (Leng et al. 2013). The anisotropic voxel dimensions and structural discontinuity in such data often result in stepped isosurfaces and other problems during 3D reconstruction. For 3D structural reconstruction, we must interpolate several intermediate slices in order to obtain volume image data with isotropic dimensions (Leng et al. 2013). When a transformation is applied to the entire image, it is referred to as a global transformation, while a transformation that is applied to parts of the image is called a local transformation (Maintz and Viergever 1998). In this case, a global image registration was performed, where the transformation was applied to the entire image.

### Anatomical landmarks used for registration

**Fig. 4:**
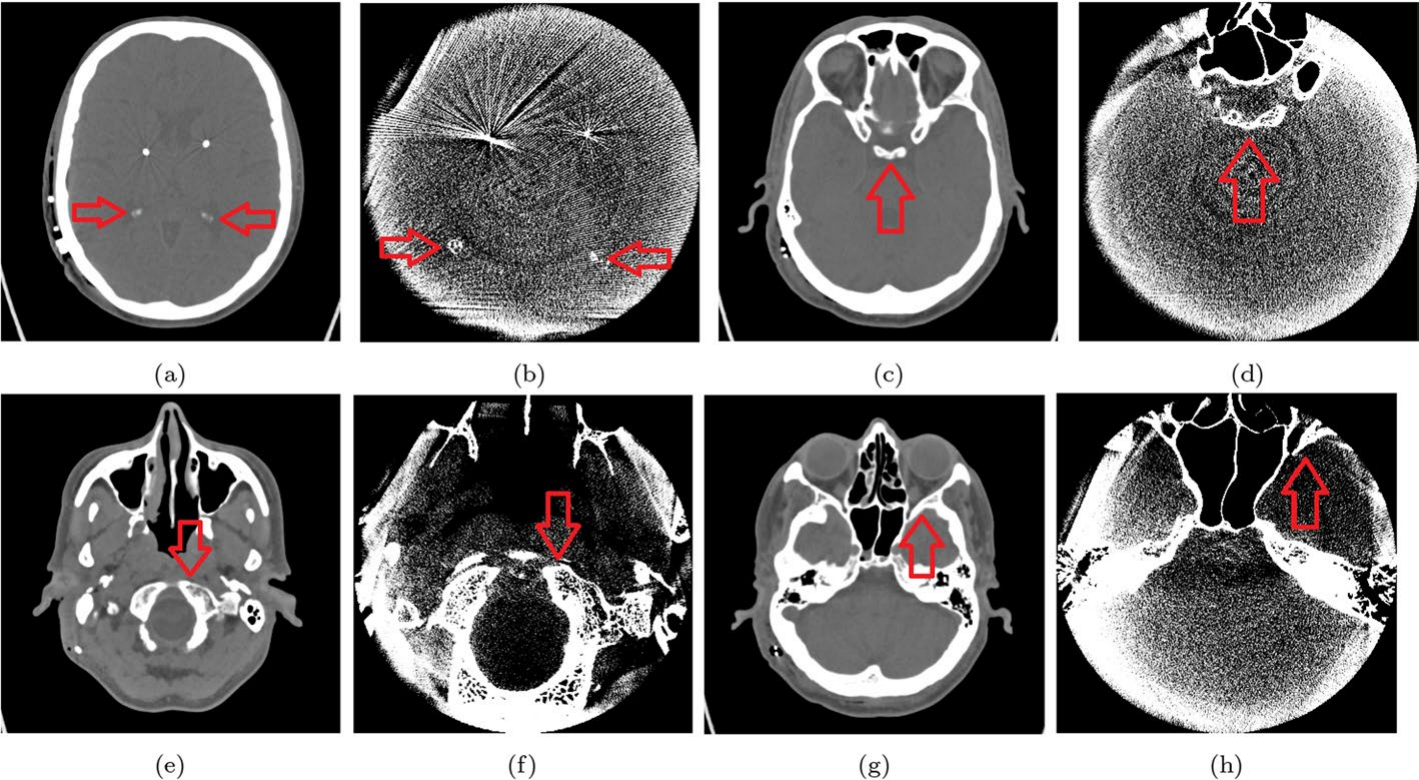
Overview of the crucial anatomical structures corresponding in both modalities: CT and DCTm images. These points are used for initiating and evaluating registration with input rater, but not the same points that were used for initialization are used for computing the target registration error (TRE). ((1) and (2)): bilateral calicifications in choroid plexus (not apparent in all patients), ((3) and (4)): clivus/tuberculum sellae, ((5) and (6)): atlantoaccipital joint, ((7) and (8)): superior orbital fissure

This work used a multimodal registration in which both images have a different FOV, which resulted in DCTm not being able to display the cap of the skull. The general shape of the medical images in these two modalities is different, as they have an oval shape in CT, while they have a round shape in DCTm/FDCT. For these reasons, we depended on the help of a physician in the field to find common landmarks for both image modalities. In collaboration with the University Hospital Magdeburg, a physician identified some of these landmarks, e.g., the semicircular canals, clivus, and the petrous bone. Figure 4 shows examplary landmarks that were used.

### Registration framework

#### Manual initialization

The anatomical landmarks are points of visual anatomy that are salient and easily located by the user (Maintz and Viergever 1998). In this work, anatomical landmarks shared between CT and DCTm images are identified interactively by the user. In the landmark-based registration, the list of identified points is sparse in comparison to the original image content, allowing for relatively fast optimization (Maintz and Viergever 1998). Furthermore, it is also affecting the runtime, initialization and the convergence to the correct minimum. A good starting point for this transformation is close to the correct solution, ensuring that it converges as soon as possible. A problem-specific approach often yields better results than a generic approach (Beare et al. 2018; Yaniv et al. 2018). In this registration with manual initialization, the user identified three corresponding points in the CT and DCTm images, and the identification of these points was performed by two users for each patient image dataset.

#### Registration components

A multi-resolution rigid registration method is based on mutual information. The registration configuration which was used in this work, had the following parameters:

In order to develop our registration workflow, we used the Simple ITK toolkit, which consists of several components that are listed below:

**Transformations**: ITKv4 provides a framework for treating both fixed and moving images in the same manner, rather than treating them differently as in classical registration approaches. The virtual image domain, a third coordinates system, is included to accomplish this. According to the ITKv4 (Yaniv et al. 2018) registration framework consist of three transformations. From the fixed image domain to the moving image domain, points are transformed as follows:

2 $$\begin{aligned} {}^M\textbf{p} = T_{opt}(T_m(T_f^{-1}(^F\textbf{p}))), \end{aligned}$$

where $T_{opt}$ represents the maps points from the virtual image domain to the moving image domain using a moving initial transform, and updated through the optimization. $T_f$-Maps virtual image points domain to fixed image points domain. $T_m$-Maps virtual image points domain to moving image points domain.

**Similarity metric:** The similarity metric used in this study was mutual information (Mattes MI), which was configured with the following parameters: number of histogram bins set to 50, random metric sampling strategy, and a metric sampling percentage of $1\%$.

**Interpolator:** Although there are numerous interpolation methods, but the method used in this study is linear interpolation because it is providing a compromise between computational efficiency and accuracy (Boyd et al. 2006).

**Optimizer:** The optimization method used is gradient descent, with the following parameters: a learning rate of 1.0, which determines the step size in parameter space along the traversal direction, an optimizing algorithm based on gradient descent is implemented. Each iteration updates the current position in accordance with:

3 $$\begin{aligned} p_{n+1}=p_n + learning\,Rate\frac{\partial f(p_n)}{\partial p_n} \end{aligned}$$

The number of iterations was set to 100, which represents the maximum number of iterations. To ensure convergence of the similarity metrics estimated in the given window size, a convergence minimum value of 1e−6 was used to confirm convergence in the energy profile of the similarity metric. The energy profile of the similarity metric was estimated using a convergence window size of 10, which corresponds to the number of values of the similarity metric.

**Fig. 5:**
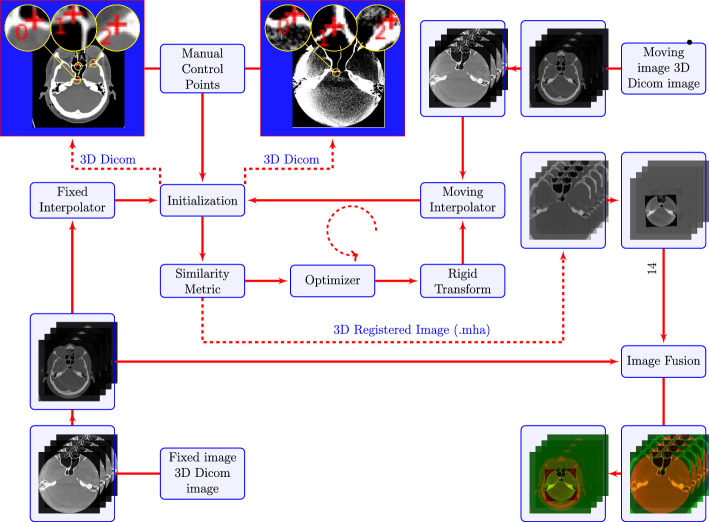
The framework of 3D multimodal medical image registration

## Results

### Visualization of registration results

**Fig. 6:**
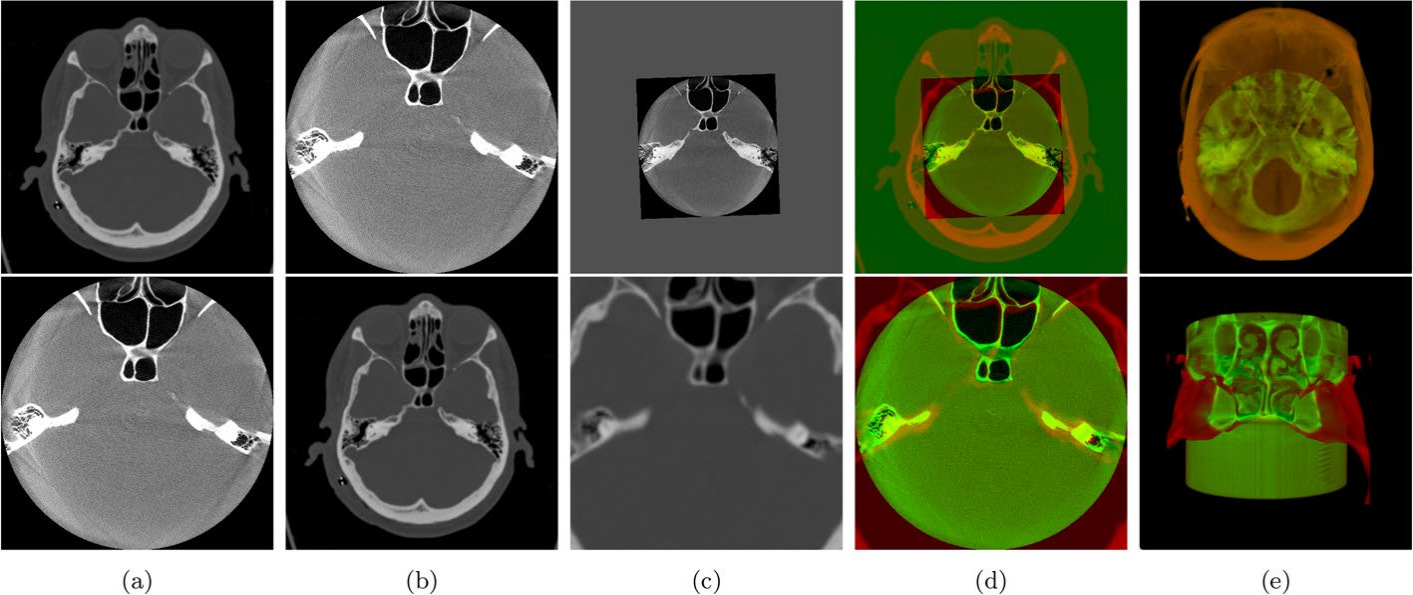
Representative images for a patient’s CT and DCTm in unregistered, registered, and fused form: **a** fixed images, **b** moving images, **c** registered images, **d** fused images, and **e** 3D fused images

Visualizing the image registration results is essential for analyzing the output. It allows us to observe the alignment of anatomical structures in both CT and DCTm images after fusing the resulting images. This alignment is clearly depicted in the Fig. 6, which displays in 6a the fixed images input, in 6b the moving images input, in 6c the registered moved images, in 6d the fused images of the fixed input images and registered images, and in 6e a 3D view of the fused images. Figure 6 shows the fixed images as source images, while the moving images are the images that need to be resampled and registered according to the source images. In addition, a well-performed registration between CT and FDCT is visualized and shown in an anatomical context. Table 1 provides information on the pixel values and pixel spacing of each image before and after registration.

**Table 1:** Representative example of images before and after resampling for one patient using CT and DCTm modalities, when we take one of these modalities as a fixed image and the other as a moving image, and vice versa

Patient	Modality	Before resampling		After resampling	
		Num voxel	Voxel size (mm)	Num voxel	Voxel size (mm)
P1	CT (fixed)	512$\times$512$\times$197	0.38 $\times$ 0.38 $\times$ 0.7	512 $\times$ 512 $\times$ 197	0.38 $\times$ 0.38 $\times$ 0.7
	DCTm (moving)	512 $\times$ 512 $\times$ 497	0.197 $\times$ 0.197 $\times$ 0.197	512 $\times$ 512 $\times$ 197	0.38 $\times$ 0.38 $\times$ 0.7
P1	CT (moving)	512 $\times$ 512 $\times$ 197	0.38 $\times$ 0.38 $\times$ 0.7	512 $\times$ 512 $\times$ 497	0.197 $\times$ 0.197 $\times$ 0.197
	DCTm (fixed)	512 $\times$ 512 $\times$ 497	0.197 $\times$ 0.197 $\times$ 0.197	512 $\times$ 512 $\times$ 497	0.197 $\times$ 0.197 $\times$ 0.197

### Results evaluation

**Fig. 7:**
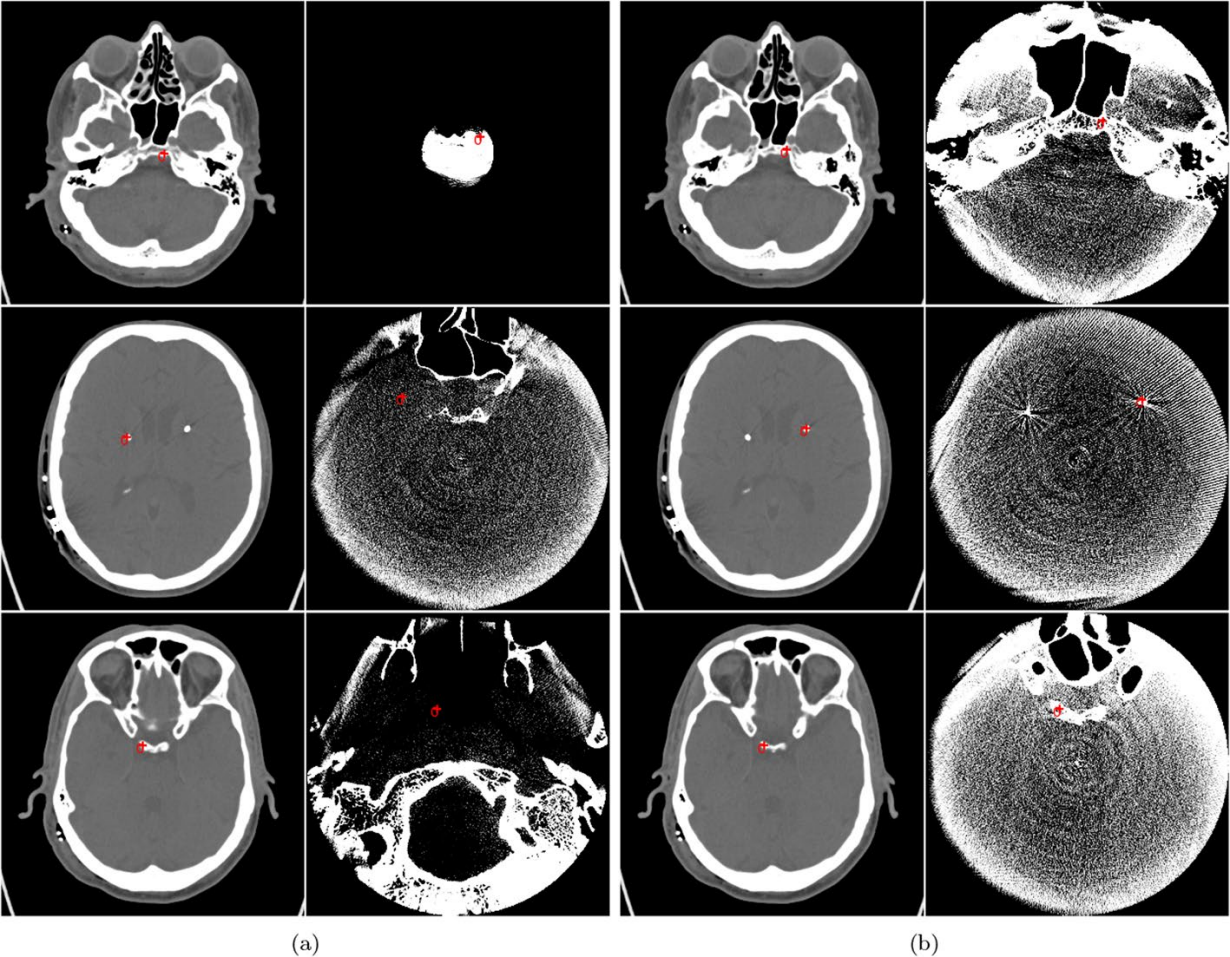
This figure illustrates the prediction value using linked cursors. The red points in between each subfigure indicate the corresponding link points between CT and DCTm images. The subfigures are arranged from left to right as follows: **a** CT input images and unregistered DCTm images, and **b** CT input images and registered DCTm images

In order to provide a comprehensive assessment of the registered images results, we used a combination of quantitative and qualitative evaluation. For qualitative evaluation, we employed various techniques, including the linked cursors approach that displays corresponding points between both images pre- and post-registration images (as shown in Fig. 7), checkerboard pattern, and image fusion via alpha blending. All of these methods yielded favorable outcomes, which are elaborated upon in more detail in “Qualitative evaluation” section. In this context of quantitative assessment, we utilized TRE. The results are presented in Fig. 12 and Table 2, which demonstrate a significant error reduction post-registration. Furthermore, the convergence of results was achieved from inputs of two users with diverse training backgrounds.

#### Qualitative evaluation

The registration results were evaluated qualitatively by overlaying the CT with DCTm images after registration and several ways exist to superimpose two (partially) overlapping images. Among the most common approaches are:

#### Approach based on linked cursors

After registration by aligning of the two volume images. Using a linked cursor approach, the clicking on one image will activate the corresponding point to be appearing on the other image. This can be one of the ways to qualitatively evaluate the result, as shown in Fig. 7.

#### Checkerboard pattern

**Fig. 8:**
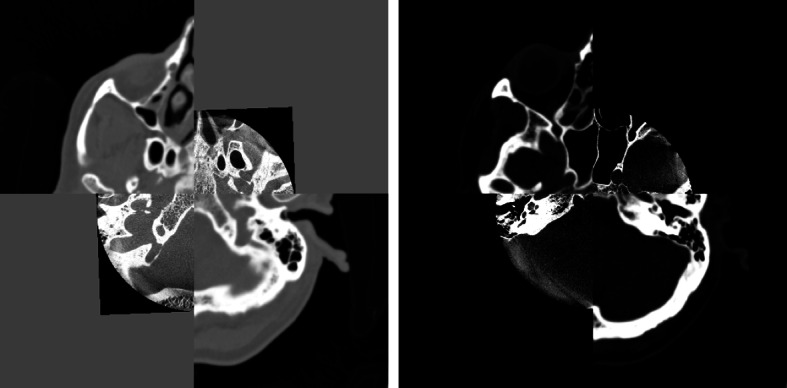
Visualization of the data after registration using a checkerboard with original pixel intensities, and rescaled pixel intensities

**Fig. 9:**
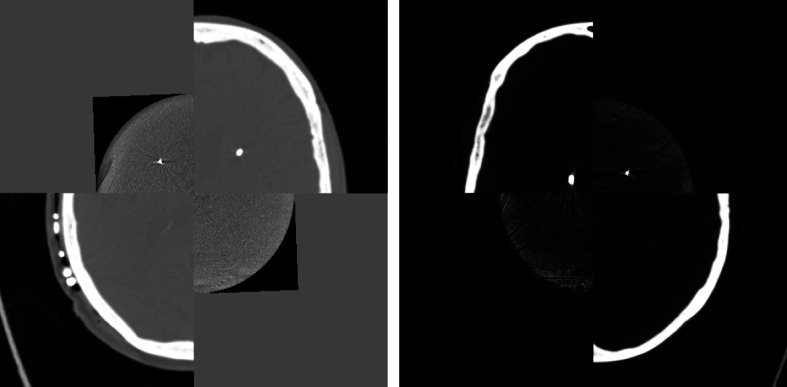
An example of CT weighted DynaCT images registered using mutual information. The two registered images are shown interleaved in a checkerboard pattern

Merging starts with loading two images whose contents fortunately overlap in physical space, this is especially evident in the background, as both images contain air (Beare et al. 2018; Yaniv et al. 2018). Figures 8 and 9 show fused images with checkerboard patterns before and after rescaling the image intensities. The checkerboard pattern is used as a valuable tool for qualitatively assessing multimodal medical image registration FDCT with CT images. Figure 8 demonstrates the assessment with anatomical context. Figure 9 shows the assessment to the electrode context. The checkerboard pattern emphasizes and enables visual verification of registration accuracy. This qualitative assessment, supported by the checkerboard pattern, improves the overall visualization of multimodal medical image registration in both anatomical and electrode contexts.

#### 3D-visualisation of segmented contacts after registration

**Fig. 10:**
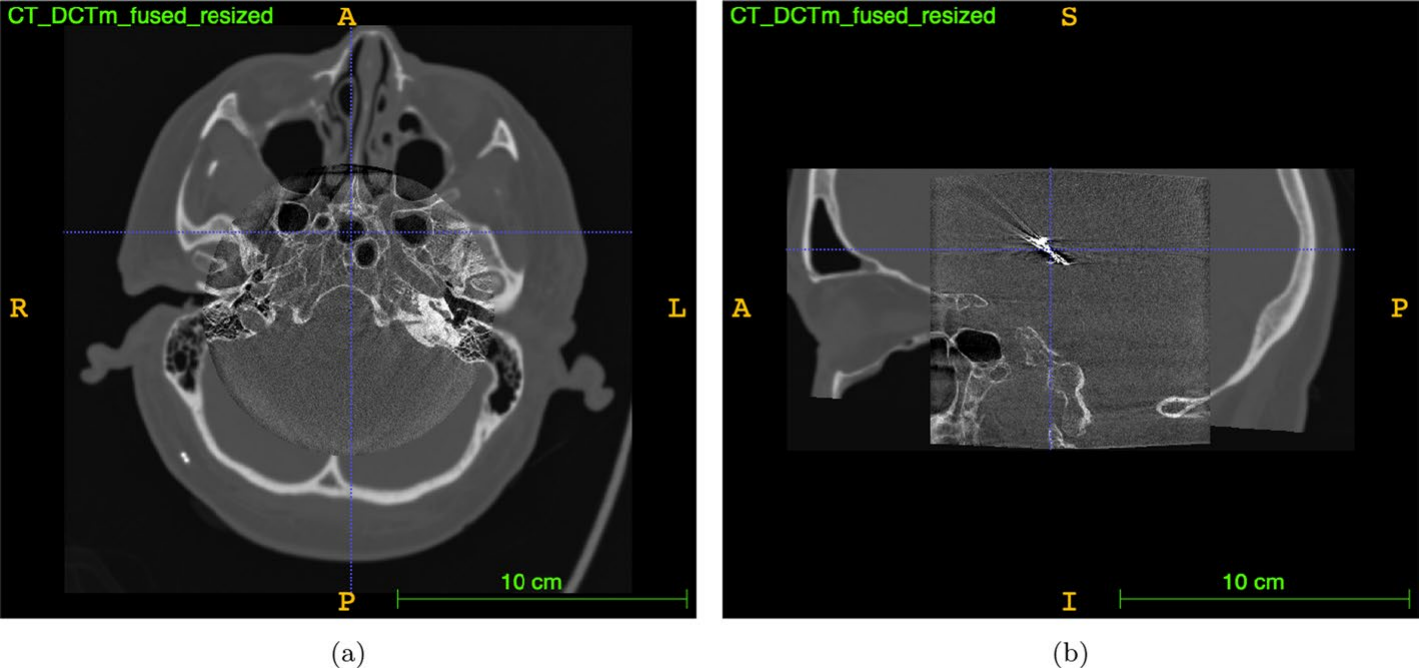
Fusion of both modalities after cutting out CT on voxels where DCTm intensities are over zero: **a** image shows axial slices and **b** image shows sagittal slices

To achieve the 3D visualization of segmented contacts on the electrodes, based on our initial hypothesis. Our aim was to investigate the capability of aligning high-resolution FDCT images, featuring voxel sizes below 0.2 mm and a limited FOV, with conventional multislice-CT images. This alignment establishes anatomical context for fusion with MRI. As illustrated in Fig. 10, the axial and sagittal views demonstrate precise anatomical alignment between CT and FDCT images.

**Fig. 11:**
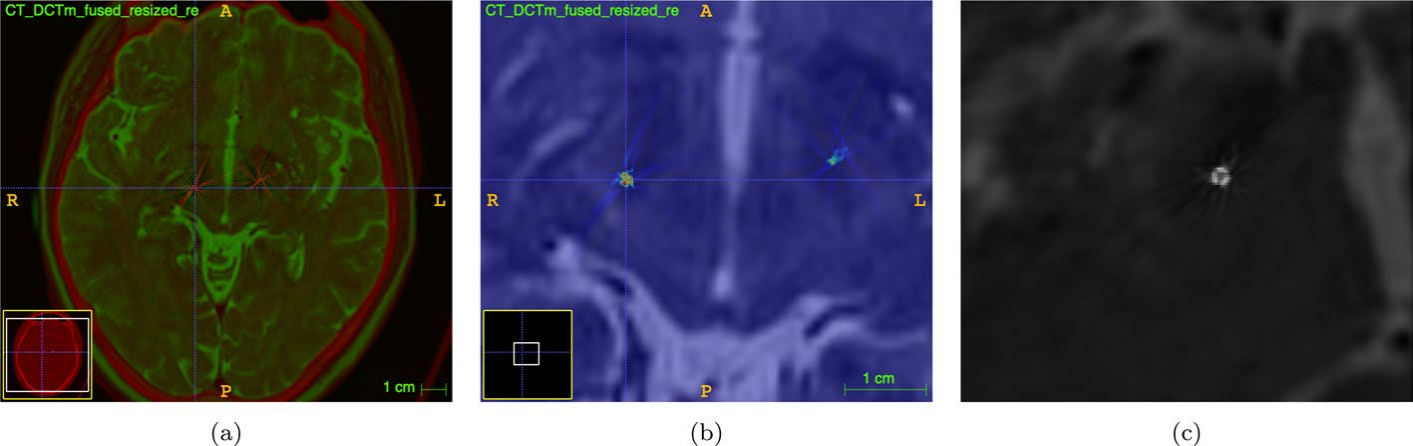
Demonstration of registration to MRI as possible postprocessing-step in future work (mean TRE: 1.7 mm, STD: 0.93 mm using ITK-SNAP Yushkevich et al. 2006), **a** CT is displayed in red and T2-weighted MRI in green, **b** segmented contacts of right electrode in red according to a hsv color-map, **c** after multi-planar-reconstruction (MPR) along an electrode based normal vector: the three segmented contacts can now be clearly delimited in grayscale color map

For clarity, we exemplified the process by automatically fusing one resulting image (a fusion of CT and FDCT images) with its corresponding preoperative T2-weighted MRI using an open-source tool, as shown in Fig. 11. This step aimed to evaluate the 3D visualization of segmented contacts in dDBS electrodes. Our overall findings suggest the feasibility of visualizing these segmented contacts, exemplified in Fig. 11c, where the three segmented contacts of the electrodes are clearly visible.

#### Quantitative evaluation

**Fig. 12:**
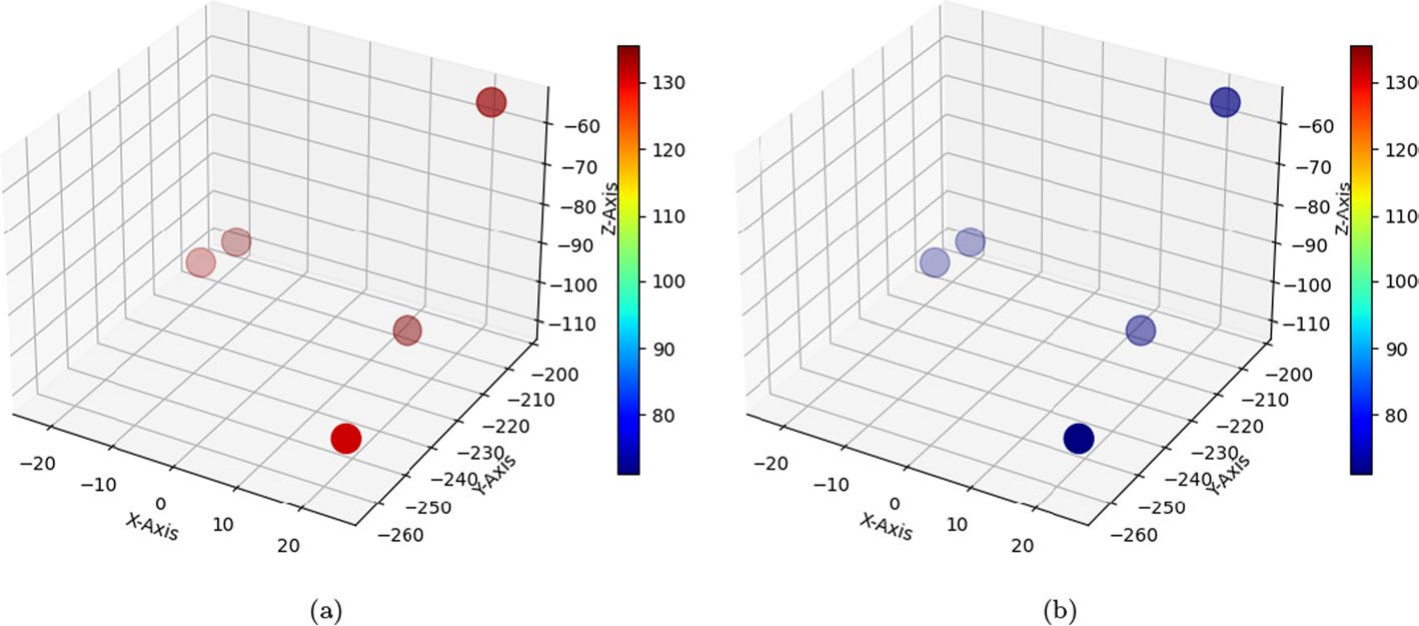
Illustration of the five points utilized by the rater to determine corresponding points between the anatomical structures in both CT and DCTm images. These points are solely used for evaluation and are distinct from the initialization points used for user input in the semi-automated registration process. The figure also presents the calculation of the TRE between the corresponding points in both image modalities, as shown in **a** for TRE points before registration and **b** for TRE points after registration

**Fig. 13:**
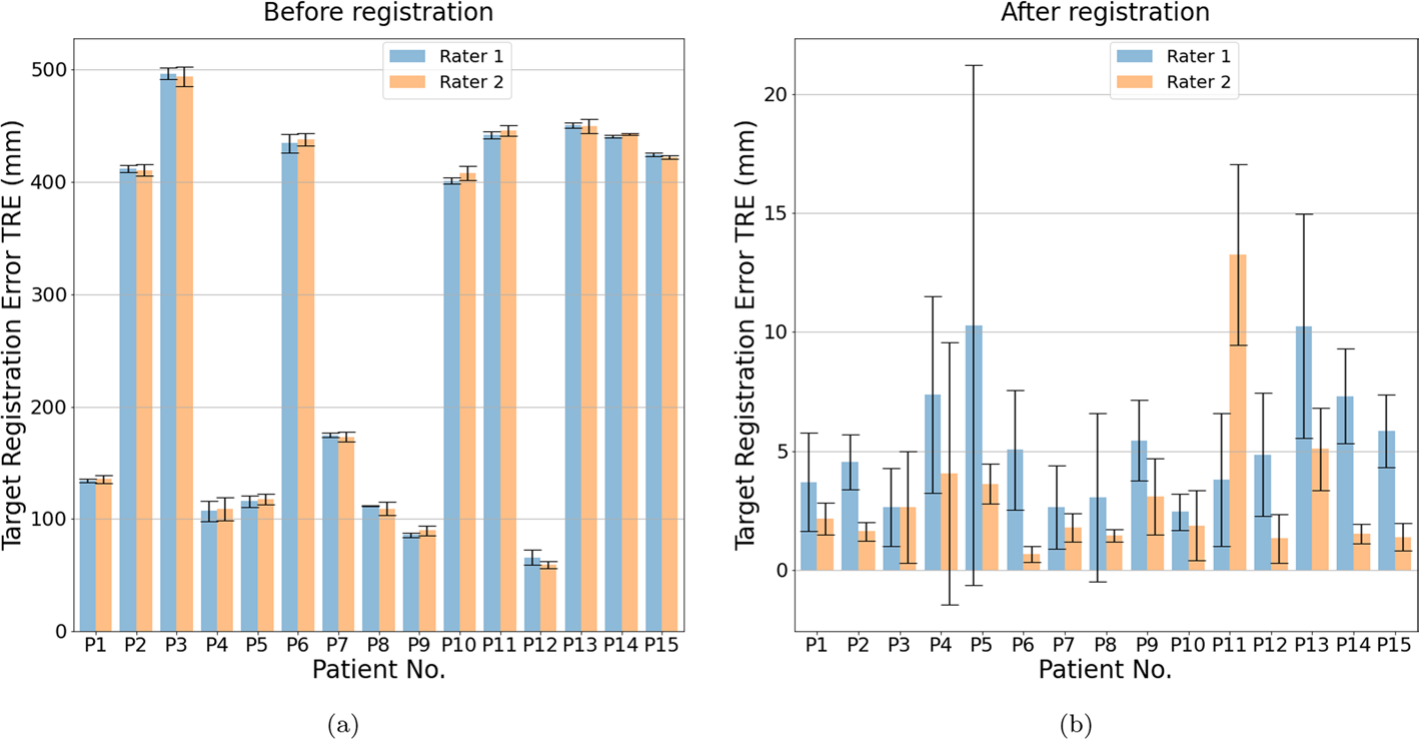
Quantitative evaluation based on mean and standard deviation in calculating TRE for five points in each patient image dataset, comparing CT and DCTm images for fifteen patient datasets with deep brain stimulation. Two raters evaluated each dataset, identifying corresponding anatomical landmarks in both image modalities through qualitative input. The TRE was calculated using a semi-automatic approach, with the first rater’s input shown in blue and the second rater’s input in orange. The figure is divided into two parts, with part **a** representing the dataset before registration and part **b** showing the dataset after registration

The performance of the registration based on quantitative evaluation has been evaluated by two assessors with different backgrounds. On a dataset of 15 patients of multimodal medical images (CT, FDCT as DCTm) with anatomical information and electrode-specific information. Figures 12, 13, and Table 2 provide an overview of TRE’s descriptive statistics.

The results of our multimodal image registration are promising and reveal substantial improvements in the accuracy of image alignment between CT and FDCT images. For further details of our results.

The evaluation of registration in the Fig. 12 depicts the TRE before in 12a and after in 12b of the five points in 3D coordinates for one patient, the corresponding points on CT and FDCT images. The red points in 12a represent high TRE before registration, while the blue points in 12b signify a significant reduction in TRE after registration. Figure 13: Presents the mean and standard deviation of five points for the evaluation of registration across fifteen patients. in 13a highlights the initially high TRE values before registration, while in 13b showcases a considerable improvement post-registration, with reduced TRE values. Additional file 1: Table S1: Provides exact values chosen by Rater 1 and Rater 2 for TRE before and after registration. Notably, there is a consistent and significant improvement in reducing TRE values after registration. Table 2: Illustrates the mean and standard deviation calculated from the five points in Additional file 1: Table S1. This summary showcases an overview of results before and after registration for two raters across fifteen patients, emphasizing the substantial improvement achieved.

The mean and standard deviation analysis in the Table 2: Shows an overview of mean and standard deviation values, providing a concise summary of the entire evaluation. There are differences between Rater 1 and 2 as can be visually derived from Fig. 13b. Precisely the average TRE for Rater 1 was 5.28 mm whereas the average TRE for Rater 2 was 3.04 mm. Additional file 1: Table S1: Specific values of the five points selected by each rater further emphasize the significant reduction in TRE after registration, reinforcing the effectiveness of the image registration process.

The 3D visualization of segmented contacts in 11c: Demonstrates the 3D visualization of segmented contacts in dDBS electrodes. The clarity of these visualizations indicates the success of our multimodal image registration in enhancing precision.

**Table 2:** Mean and standard deviation of the target registration error computed for each pair of the DCTm and CT scan images, before and after registration in the proposed method, via evaluation of the rater 1 and rater 2 for each of them

NO.	TRE before registration (mm)				TRE after registration (mm)			
	Rater 1		Rater 2		Rater 1		Rater 2	
	Mean	SD	Mean	SD	Mean	SD	Mean	SD
Patient 01	133.88	1.50	135.56	3.35	3.70	2.08	2.15	0.66
Patient 02	411.65	3.16	410.48	5.11	4.54	1.14	1.63	0.39
Patient 03	496.36	5.35	493.61	9.00	2.65	1.64	2.64	2.35
Patient 04	107.01	8.88	108.60	10.21	7.38	4.14	4.06	5.51
Patient 05	115.85	5.08	117.72	4.60	10.29	10.93	3.62	0.84
Patient 06	434.27	8.41	437.35	5.52	5.05	2.51	0.67	0.34
Patient 07	174.56	1.84	173.10	4.07	2.64	1.75	1.79	0.59
Patient 08	111.39	0.43	109.28	5.99	3.04	3.54	1.44	0.26
Patient 09	85.71	1.74	89.65	4.62	5.44	1.70	3.09	1.61
Patient 10	400.80	2.83	407.94	6.19	2.44	0.77	1.87	1.47
Patient 11	441.43	3.08	445.56	5.06	3.78	2.8	13.26	3.79
Patient 12	65.75	6.78	59.22	3.43	4.85	2.59	1.32	1.02
Patient 13	450.30	2.23	449.36	6.31	10.26	4.71	5.08	1.73
Patient 14	440.19	1.12	442.64	0.75	7.31	2.0	1.53	0.4
Patient 15	424.37	1.4	422.02	1.58	5.83	1.53	1.39	0.59

## Discussion

To the best of our knowledge, the present work is the first to three-dimensionally visualize the segmented contacts of directional DBS electrodes in postoperative CT for 15 different Parkinson’s patients. The background of the present work is that conventional imaging (MRI, CT) in DBS is not able to visualize the segmented contacts relevant for the control of the electric field. This is mainly due to the occurrence of metal artifacts and insufficient spatial resolution. The visualization of the segmented contacts in the target area of brain stimulation has the potential to increase procedural safety with regard to a shorter operation time (Schmidt et al. 2022). The solution to visualize the inner electrode structure was to use a postoperative DynaCT with advanced metal artifact reduction. However, because MRI (depicting the stimulation target) and DynaCT (depicting the electrode) represent different amounts of anatomical information with significantly different contrasts it was decided to use a routine postoperative CT scan for generating a CT-DynaCT fusion image. In that way we expand the narrow FOV of the DynaCT and thus provide additional anatomical information for matching it to the preoperative MRI in future work. Since image fusion of CT to preoperative MRI in general has already been introduced (Studholme et al. 1996; Hille et al. 2015; Al-Saleh et al. 2016) the method workflow presented here may be seamlessly integrated to existing DBS imaging workflows. In the context of DBS image fusion of intraoperative FDCT to preoperative MRI during implantation has been shown to be applicable without additional microelectrode recordings (MER) (Soler-Rico et al. 2022). Furthermore measuring the electrodes’ orientation properties via rotational fluoroscopy and fusion of FDCT to stereotactic CT and preoperative MRI respectively has been introduced by Egger et al. (“iron-sight method”) (Egger et al. 2022). To our knowledge those approaches for fusing CT/FDCT to MRI relied on images depicting the whole skull. Coming back to specifically 3D-visualizing the electrodes’ segmented contacts in anatomical context these images hardly resolve the demanded structures though information about orientation is exploitable (Hellerbach et al. 2018). There are low records of specifically fusing spatially highly resolved images with limited FOV in the required multimodal manner. Decent DCTm/MRI-fusion results have been obtained via region-based registration of spine images (Hille et al. 2015) where the FOV was restricted to the same vertebra in both DCTm and MRI thus sharing a comparable amount of complementary information. Unfortunately, the intracranially poorer soft tissue contrast as well as a more narrow FOV in DCTm compared to conventional neuro-CT lead to hardly any corresponding anatomical features with MRI. Thus, as pointed out in the method section, a direct approach for fusion of DCTm to MRI did not seem promising. As imaging in DBS involves pre- and postoperative CT examinations, registration of CT to DCTm could be used as means to transfer highly resolved electrode-related information of DCTm to MRI. That is either because the target CT is already registered to the MRI or because fusion to MRI via established commercial or open source tools could be following.

Based on this hypothesis, we aimed to investigate the ability of registering spatially highly resolved FDCT images with voxel sizes below 0.2 mm and a cut-out FOV to conventional multislice-CT images providing anatomical context for fusion to MRI as depicted in Fig. 10. Additionally, for demonstration purposes, we automatically fused one resulting image to its corresponding preoperative T2-weighted MRI using an open source tool (Yushkevich et al. 2006). This had been done for testing the 3D-visualization of the segmented contacts within the target organ. Generally, the results indicate that it is indeed possible to visualize these segmented contacts, as depicted in Fig. 11. A semi-automated (user-initialized) anatomical landmarks approach was utilized to register the multimodal images. The registration was initialized separately by two users with different training backgrounds, as was quantitative evaluation using TRE. As a result of the two users’ inputs, the TRE’s from Rater 2 were lower, as shown in Fig. 13. Since Rater 2 is a doctor trained in neuroanatomy, it becomes clear initialization and evaluation using TRE require a user with knowledge of brain anatomy on CT and FDCT images. This result underscores the pivotal role of the quality of user input in semiautomated registration and evaluation. Since images depicting the skull to its full extent may not exclusively be generated via CT but also via FDCT the proposed method should theoretically be applicable intraoperatively when a multi-purpose x-ray system is available. The proposed method is currently not suitable for applying it in the operating room and thus several limitations have to be overcome. Firstly, the magnitude of the average mean TRE around 3.5 mm seems to high for a clinical application. Secondly, the semi-automated approach is robust and accurate but time consuming when it comes to choosing appropriate landmarks. Thirdly, a single fusion took about ten minutes which seems not acceptable during a procedure. Besides, postoperative CT-images were used though preoperative CT scans were availabe. This had been done to minimize the expense of interpolation as the postoperative images provided the finest slice thickness. Generally using lower slice thicknesses will improve the results of registration (Xu et al. 2017).

Additionally our study did not specifically address the matter of differences in radiation exposure between CT and hrFDCT. Potentially this could lead to disadvantages for patients and thus should be investigated in future research. FDCT may show local maxima in skin dose (Schegerer et al. 2013) but generally exhibits dose magnitudes comparable to it’s corresponding conventional CT examination (Struffert et al. 2015). It has to be pointed out effective dose magnitudes underlay several factors for instance scan parameters, reconstruction kernel and the patients’ physical constitution in particular.

In terms of computing resources, the used RAM in this study was 32.0 GB and more details can be found in this “Computing resources and toolkits” section. The study used a dataset consisting of fifteen patients obtained from a single center. However, to improve the research, it is recommended to use datasets from different clinics and centers with varying scanners to increase the variety and size of the dataset.

## Conclusion and future work

Our study demonstrated the feasibility of using semi-automated registration of FDCT to CT to generate a CT-like image viewing the DBS electrodes segmented contacts that may easily incorporated to established CT-MRI fusion workflows. Whether the proposed method combined with fusion to MRI really could shorten the time for postoperative adjustment of stimulation parameters or be integrated in existing orientation estimation workflows (Hellerbach et al. Nov. 2018) may be subject to future research as stated previously. For instance a faster and fully automated registration between multimodal images could be explored using threshold-based segmentation or a supervised convolutional neural network based on the data from the current study. Furthermore verifying the most effective combination of optimizers, similarity metrics, and interpolators in order to reduce the target registration errors, thus improving the accuracy of the multimodal image registration to yield a TRE not higher than 1 mm (Al-Jaberi et al. 2023). Furthermore, as a future direction, it would be worth investigating whether a more narrow FOV of the FDCT (with smaller voxel sizes) could be employed instead of the current FOV to improve the spatial resolution of segmented contacts of DBS electrodes and increase the clarity of 3D visualizations. Ultimately determining the accuracy on estimating lead orientation ought to be explored. Overall, these results provide valuable insights to the field of multimodal imaging, offering a reliable method for enhancing the precision of image registration in medical applications. Our image registration technique effectively reduces registration errors, as evidenced by the consistent improvement in TRE values across patients and raters. The mean and standard deviation provide a comprehensive overview of the reliability and stability of our results, ensuring the robustness of our methodology. The 3D visualizations of segmented contacts in dDBS electrodes further underscore the practical significance of our findings, highlighting improved accuracy in aligning CT and FDCT images. Our study provides valuable insights into the use of multimodal image registration to visualize DBS electrodes and could have significant implications for enhancing surgical routines and thus ultimately patients’ quality of life.

### Supplementary Information

**Additional file 1.** The supplementary information provided alongside our study on image registration and fusion of CT and hrFDCT offers valuable insights into the qualitative and quantitative evaluations conducted. Comprising three figures and a table, these materials enrich the understanding of our findings. the supplementary materials accompanying our study provide additional qualitative and quantitative evaluations, enhancing the robustness and applicability of our findings in the context of image registration and fusion in medical imaging.

## Data Availability

The datasets used and/or analyzed during the current study are available from the corresponding author on reasonable request.

## Abbreviations

AD: Alzheimer’s disease
FDCT: Flat detector computed tomography
CT: Computed tomography
DBS: Deep brain stimulation
DCTm: DynaCT micro
DICOM: Digital imaging and communications in medicine
FOV: Field of view
GD: Gradient descent
IP: Image processing
IR: Image registration
ITK: Insight toolkit
MI: Mutual information
MHA: MetaImage
MIR: Medical image registration
MRI: Magnetic resonance imaging
PD: Parkinson’s disease
RIR: Rigid image registration
ROI: Region of interest
STN: Subthalamic nucleus
TRE: Target registration error
