# Supplementary material for: 3D-visualization of segmented contacts of directional deep brain stimulation electrodes via registration and fusion of CT and FDCT
Source: EJNMMI Rep. 2024 Jun 14;8(1):17. doi: 10.1186/s41824-024-00208-6 (PMC11286893; doi:10.1186/s41824-024-00208-6)
Supplement: Supplementary file 1 — Additional file 1. The supplementary information provided alongside our study on image registration and fusion of CT and hrFDCT offers valuable insights into the qualitative and quantitative evaluations conducted. Comprising three figures and a table, these materials enrich the understanding of our findings. the supplementary materials accompanying our study provide additional qualitative and quantitative evaluations, enhancing the robustness and applicability of our findings in the context of image registration and fusion in medical imaging. [file 41824_2024_208_MOESM1_ESM.pdf]

Supplementary information

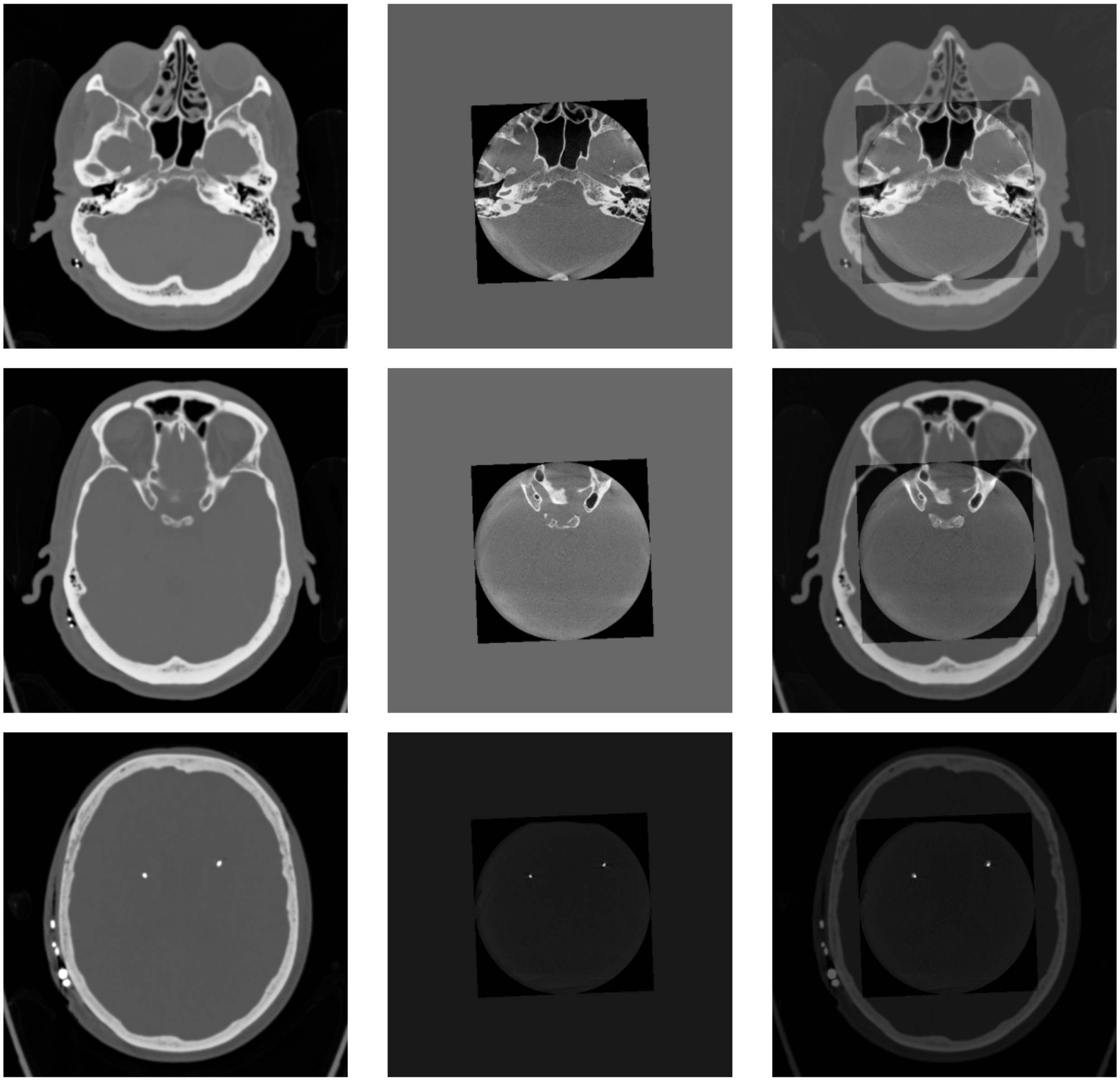

(a) (b) (c)

Figure S1: The Figure illustrates the application of CT images as a fixed reference image and DynaCT images as a moving image in the context of multimodal image registration. Specifically, (a) Depicts the CT image, (b) Displays the DynaCT image after registration, and (c) Demonstrates the fused image of both CT and DynaCT images.

Table S1: Using five corresponding points identified by rater 1 and rater 2, the target registration error between these points in the DynaCT and CT images is calculated.

	TRE before registration(mm)										TRE after registration(mm)									
	Rater 1					Rater 2					Rater 1					Rater 2				
	P1	P2	P3	P4	P5	P1′	P2′	P3′	P4′	P5′	P1	P2	P3	P4	P5	P1′	P2′	P3′	P4′	P5′
Patient 01	134.0	131.19	135.02	133.7	135.51	136.81	139.39	138.43	131.65	131.52	6.35	5.88	2.39	0.98	2.88	2.09	1.53	3.35	2.19	1.57
Patient 02	413.52	416.85	408.51	410.67	408.72	407.13	405.95	416.59	416.84	405.91	4.14	3.83	3.34	6.63	4.74	1.16	1.16	1.91	1.79	2.11
Patient 03	497.69	491.49	490.83	496.13	505.68	490.22	491.98	498.4	480.17	507.28	1.92	1.61	2.87	5.73	1.12	0.92	1.62	2.21	7.26	1.17
Patient 04	124.51	103.9	100.72	101.24	104.69	123.26	118.15	100.52	97.37	103.7	6.02	2.95	13.68	3.62	10.62	1.73	15.08	0.97	1.22	1.31
Patient 05	112.9	123.79	115.7	108.65	118.19	112.44	120.77	113.74	116.75	124.91	2.13	31.61	9.44	4.47	3.82	3.9	3.07	4.75	4.06	2.31
Patient 06	419.24	441.72	436.62	431.76	442.02	439.92	431.37	430.18	443.71	441.58	5.14	2.41	5.18	2.98	9.55	0.45	0.45	0.79	0.4	1.29
Patient 07	176.05	171.2	174.24	176.3	175.02	172.26	178.25	166.4	172.31	176.28	0.61	5.75	1.98	3.16	1.69	1.73	2.47	2.13	0.71	1.88
Patient 08	112.07	111.35	111.21	110.77	111.5	112.34	104.74	112.09	100.21	116.99	10.04	1.13	1.15	2.25	0.61	1.59	1.61	1.2	1.73	1.06
Patient 09	84.82	88.65	84.01	86.71	84.3	90.34	82.89	86.5	96.26	92.28	3.98	5.41	8.65	5.11	4.06	0.99	3.51	1.67	3.77	5.53
Patient 10	398.49	398.89	406.23	399.51	400.89	404.93	403.01	416.79	401.19	413.8	2.25	1.05	3.24	2.75	2.9	1.5	4.78	0.81	1.2	1.09
Patient 11	437.97	437.39	443.56	443.7	444.52	444.48	453.58	437.69	446.28	445.79	0.62	3.87	5.35	8.11	0.96	9.47	17.83	14.35	8.22	16.44
Patient 12	74.71	71.36	59.08	57.2	66.43	55.41	56.69	57.63	61.76	64.6	0.81	0.37	1.31	3.26	0.85	2.21	6.57	5.43	8.43	1.62
Patient 13	448.7	448.21	449.4	450.78	454.41	444.44	439.68	456.08	451.71	454.88	7.1	3.24	17.03	11.74	12.18	4.79	7.71	4.05	6.17	2.7
Patient 14	440.79	441.53	440.74	438.37	439.51	441.33	443.28	443.46	442.58	442.55	10.19	6.46	4.51	8.89	6.51	2.18	1.38	1.79	1.05	1.26
Patient 15	424.62	424.97	425.34	425.31	421.63	422.79	419.72	423.5	423.53	420.57	4.94	6.38	6.32	8.03	3.47	0.25	1.72	1.37	1.71	1.89

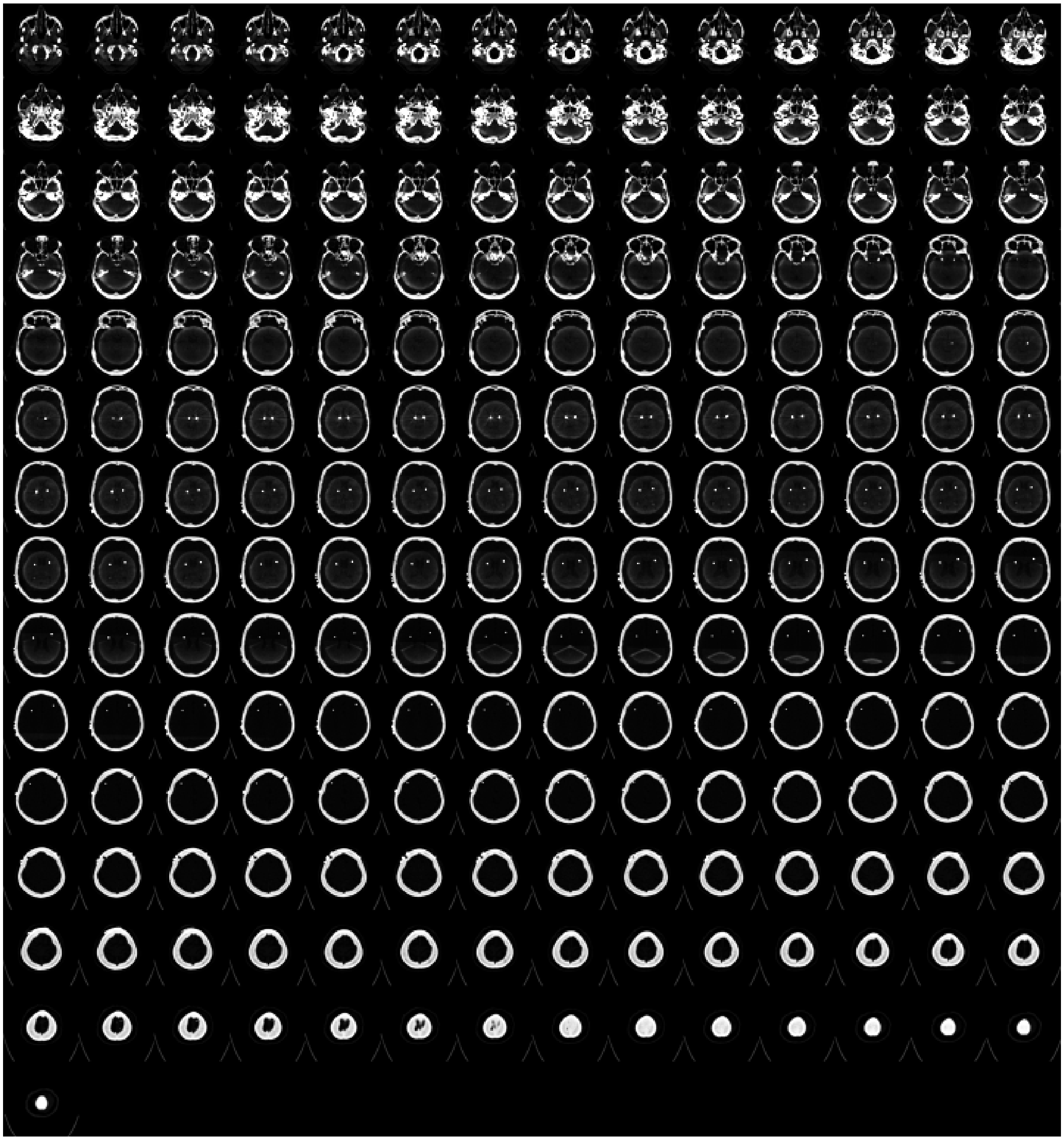

Figure S2: The Figure shows the fusion of registered DynaCT and CT images for one patient's data.

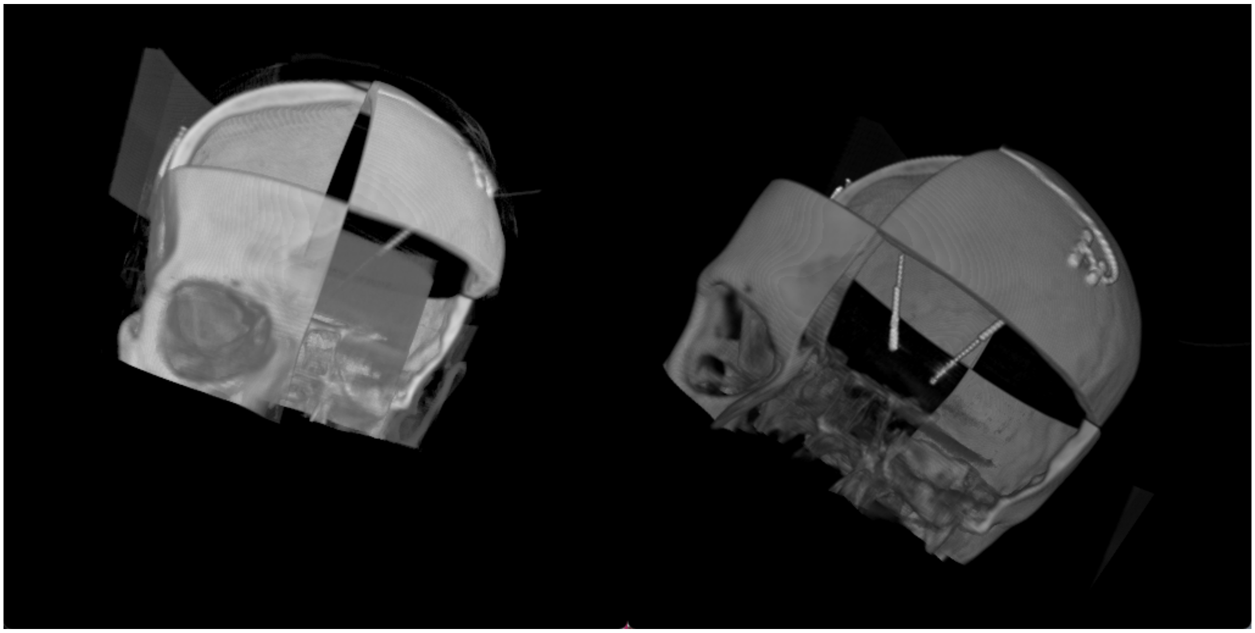

(a) (b)

Figure S3: Visualization of the data after registration using a checkerboard with original pixel intensities, and rescaled pixel intensities.
